# Moms on the move: A qualitative exploration of a postpartum group exercise program on physical activity behaviour at three distinct time points

**DOI:** 10.1080/17482631.2023.2172793

**Published:** 2023-01-29

**Authors:** Iris A. Lesser, Carl P. Nienhuis, Gillian L. Hatfield

**Affiliations:** School of Kinesiology, University of the Fraser Valley, Chilliwack, BC, Canada

**Keywords:** Postpartum, exercise, mental health, identity, physical activity

## Abstract

**Purpose:**

Physical activity (PA) after the birth of a child is associated with improved physical and mental health benefits. The aim of the study was to explore new mothers’ experiences of PA before and after participation in a group-based PA program for new mothers. The study has three research questions: how new mothers felt; 1) about PA after the birth of their child 2) about engaging in a group-based PA program and 3) after the program ended about ongoing PA engagement.

**Methods:**

We conducted an 8-week bi-weekly PA program for postpartum women. To understand the experience of postpartum women engaging in a group-based PA program we conducted one-on-one semi-structured interviews before, after, and at 6-month follow-up.

**Results:**

Of the *n* = 21 participants in the Moms on the Move study; *n* = 17 completed pre interviews.s. One primary theme emerged from pre-program interviews where mothers shared how they were lost as to where to start PA. Post-program interviews produced two primary themes; engaging in PA helped mothers, and mothers learned to rethink PA.

**Conclusions:**

Postpartum women who participated in this group-based PA program had positive benefits to their mental and physical health and were able to alter their PA behaviour.

## Introduction

The postpartum phase can be fraught with challenges as women navigate the identity shift of motherhood while experiencing mental and physical health changes, all while caring for an infant (Deave et al., 2008;Forster et al., 2008;Homewood et al., 2009). Despite this knowledge, there is a paucity of postpartum support leaving many women disoriented about their postpartum body, often leading to mental distress (Goodman & Gotlib, 1999;Homewood et al., 2009). Physical activity has the capacity to reconnect women with their new bodies (McGannon & McMahon, 2021) while reducing mental duress (Sampselle et al., 1999) with an array of physical health benefits (DiPietro et al., 2019). New mothers state that an important factor in their postpartum care is re(gaining) health and well-being (Finlayson et al., 2020). Despite this desire, physical activity levels are lower among women who have young children (Guthold et al., 2018) and remain lower in working mothers (Limbers et al., 2020).

Mothers consistently cite a lack of opportunity to engage in physical activity due to lack of childcare, access to safe and feasible exercise programming postpartum (Evenson et al., 2014) and time constraints (Makama et al., 2021;Saligheh et al., 2016). In addition, there is low confidence around physical activity engagement in a changed body (Makama et al., 2021) and physical activity competence is further impacted as mothers experience identity conflict within the motherhood identity (McGannon & McMahon, 2018). Factors which have been shown to improve success in group-based physical activity postpartum have included a supportive, non-judgemental environment, allowance for the inclusion of children, and programming provision, which focuses on strength and function (Peralta et al., 2022). When these facilitators are at the forefront of exercise provisions, postpartum women have been shown to have positive improvements in mental well-being (Hatfield et al., 2022). However, it is unknown how women felt about starting physical activity post-birth, their experience in a socially supportive, inclusive and group-based physical activity program, and how it impacted future physical activity behaviour. Gaining insight into the experience of new mothers in this form of programming is essential to assess the impact of supportive physical activity programming postpartum on physical activity engagement. Therefore, this qualitative study aimed to provide further insight into how a group-based exercise program for new mothers may impact postpartum engagement in physical activity and adherence to physical activity. Group-based physical activity holds promise for allowing new mothers to be physically active in a supportive environment with their infant present. However, to date, group-based physical activity programming in postpartum women has yielded little success at increasing physical activity or psychological well-being (Peralta et al., 2021). Furthermore, there remains a gap in the knowledge surrounding how women feel about engaging in physical activity postpartum and whether they have the necessary education and support to do so.

While it is necessary to evaluate the impact of an intervention, given the lifelong engagement in physical activity for health and well being, longer term follow-up after programming ends is needed. Given the high attrition rate with physical activity in the general population (Dishman, 2001) and limited long-term success in postpartum physical activity interventions (Mailey & McAuley, 2014; Miller et al., 2002) it is possible that positive improvements in physical activity engagement from this form of programming is not long-lasting. Further, understanding the role of the postpartum physical activity experience on long-term physical activity engagement as mothers return to other societal roles such as employment is lacking. Mothers who return to work have a high likelihood of physical inactivity (Limbers et al., 2020), and therefore a better understanding of long-term physical activity engagement is needed. There is a need to understand the strategies which assist parents in the prioritization of personal well being and knowledge around overcoming barriers to engagement (Mailey et al., 2014).

Given the unique timeline of the postpartum phase and the knowledge that can be accrued by hearing from women at different timepoints we followed women before, after and at 6 months faftera group-based physical activity intervention. Therefore, the purpose of this study was to explore new mothers’ experiences of physical activity before and after participation in a group-based program for new mothers. The study has three research questions: how new mothers felt 1) about physical activity after the birth of their child 2) about engaging in a group-based physical activity program for new mothers and 3) after the program ended about ongoing physical activity engagement.

## Methodology

### Design

A qualitative description methodological approach was utilized, whereby researchers aimed to understand participant perspectives of the exercise program and physical activity engagement (Creswell, 2013; Grbich, 2013). Qualitative description methodologies are guided by inductive processes, which recognize the subjectivity of participant perspective, and are designed to develop an understanding of participant experience (Bradshaw et al., 2017). Intending to provide a rich description of participant experience, findings from studies that adopt a qualitative description methodological approach are often utilized by practitioners and policymakers (Bradshaw et al., 2017;Grbich, 2013). Consistent with qualitative description methodology, some measures were employed to increase the trustworthiness of the research. Rapport building, maintaining prolonged engagement through several rounds of interviews, and including participants’ voice within study findings supports research credibility in qualitative description methodologies (Finlay, 2006). Finally, participants were reminded that they were under no obligation to participate and were free to refuse to answer questions; this combined with participant informed consent were ethical checkpoints which support trustworthiness.

### Intervention

In this study, women between 6 weeks and one-year postpartum participated in a bi-weekly outdoor group exercise program for 8 weeks. The group exercise intervention was a 45-min group fitness class, Les Mills TONE^TM^, created by Les Mills International (Auckland, NZ). Classes were offered twice per week at two different times to keep group size under ten, in line with pandemic public health restrictions at the time. Participants were given the option to bring their baby to help reduce potential barriers with exercise engagement. In each class, the instructor provided options to either increase or decrease the intensity as needed. In addition, options for using the baby as weight if the mother had to be attending to the baby during exercise were provided.

### Participants

Participants who enrolled in the initial Moms on the Move study (Hatfield et al., 2022) were additionally invited to participate in three semi-structured interviews by phone or online (Zoom). Of the 21 enrolled Moms on the Move participants, 17 volunteered to complete interviews prior to the exercise program. Participants average age was 31 years old (SD = 2) with infants averaging 6 (SD = 3) months at the start of programming. Of the 17 participants, 10 gave birth via caesarean section and 7 had a vaginal birth. The majority (12 of 17) of participants reported breastfeeding at the start of the study, and the average body mass index of participants was 28.0 (SD = 5.9) kg/m^2^. Two participants did not complete the exercise program, but one of them completed a post-program interview, resulting in 16 participants. When contacted at 6-months post-program, 12 mothers agreed to participate.

### Data collection

Semi-structured interviews were conducted, and interviews took place via a university-licenced virtual platform (Zoom) (n=10) or by telephone (n=35). Telephone interviews lasted 13 to 68 min (*M* = 20 min) and zoom interviews lasted 20 to 50 minutes (*M* = 37 min) in duration. All interviews were audio recorded and later transcribed for analysis. After transcription audio files were deleted and transcribed files were stored on a password protected computer. A research assistant was trained to conduct interviews with research participants.

The interview guide was developed prior to the intervention. Before the interview participants were given a review of the purpose of the interview, and assurance of confidentiality of the interview and recording. The pre-program interview primarily explored current physical activity behaviours; the post-program interview addressed intervention impact and program experience; and the 6-month follow-up interview explored changes to physical activity and well-being since program completion (sample questions are shown inTable 1).

**Table I.** Sample interview guide questions used within the study by timeline.

Interview Guide Section	Interview guide questions
Pre-Program	Can you tell me about your experience as a parent? Can you describe your current experience with physical activity? What prevents you from being physically active? What helps you to be more physically active? What is your main motivation for being physically active?
Post Program	Can you describe your experience with the program? How would you compare the program to other physical activity programs you have done? How did your participation in the program impact how you view yourself? What significance did the other participants have on your experience in the program?
6-MonthsPost Program	Since the completion of the program what are you currently doing to look after your personal well-being? What factors do you think has influenced your ability to be or not by physically active? Since participating in the program how has your physical health changed? Overall, what significance has the program had on your life?

### Data analysis

The research team used an inductive thematic analysis (Bradshaw et al., 2017) to consolidate meaning and identify themes in how mothers described their experiences with physical activity at different time points in relation to the exercise program (Braun et al., 2016). Following the completion of each round of interviews, interviews were transcribed verbatim by a trained research assistant and subsequently uploaded to NVivo (version 11;QSR International, 2015), which was used to generate codes. Consistent with thematic analysis (Braun et al., 2016), the first phase consisted of familiarization with the data wherein two researchers independently read and reread the data. During the second phase, coding, researchers identified and labelled data relevant to the primary research questions. Given the flexible nature of thematic analysis, during the coding process some codes were adjusted, split or collapsed together to fit the developing analysis (Braun et al., 2016). An additional round of coding was conducted to develop more latent codes. Once coding was complete, a third phase of analysis occurred where nodes were clustered into patterns containing broader meanings. After initial codes were clustered into 12 different themes, codes within each theme were reviewed to ensure each theme contained a central organizing concept and to ensure identifiable distinction between themes. During this stage of review, some themes were merged and others removed as well as several subthemes were identified as highlighting important aspects within a theme. At this stage of analysis, theme definitions were developed which serve as brief description of each theme as presented within the results. Theme and subtheme names were generated reflective of participant voices. Consistent with qualitative description methodology, pseudonyms were used to protect participant anonymity and to ensure all participant voices are represented within the results (Bradshaw et al., 2017).

### Ethics approval and consent to participate

The study followed the guidelines on human medical research according to the declaration of Helsinki (World Medical Association, 2013). Institutional ethics approval was received from the University of the Fraser Valley Human Research Ethics Board prior to data collection (Ethics Approval 100,773), and participants provided informed consent orally and in writing. Study participants were volunteers and confidentiality were adhered to. Participants were informed that they could withdraw from the study at any time without any explanation and withdraw their data within one month after interviews were conducted. Transcribed interviews were given a participant code and pseudonyms were employed for publication purposes.

## Results

Results were organized into different groups of themes based on the three different stages of interviews. One primary theme emerged from pre-program interviews where mothers shared how they were lost as to where to start physical activity. This overarching theme highlights the meanings of the subthemes from pre-program data: a) the ways moms are frustrated with physical activity, b) the ways moms view themselves and c) the ways moms need help. Given similarity in data emerging from post-program and 6-month follow-up interviews, analysis produced two themes and five subthemes. The first primary theme, engaging in physical activity helped mothers, contained three subthemes: the program helped the mothers a) feel better in their bodies; b) gain benefits and c) connect with others. The second theme that mothers learned to rethink their physical activity contained two subthemes: mothers a) experienced a kickstart into physical activity and b) were challenged to maintain physical activity. A summary of themes and subthemes are displayed inTable 2.

**Table II.** Interview primary themes and subthemes.

Themes	Subthemes
**Pre-Program Data**	
Mothers were lost as to where to start physical activity again	*The way moms are frustrated with physical activity* *The way moms view themselves* *The way moms need help*
**Post-Program Data**	
Engaging in physical activity helped mothers Mothers learned to rethink their physical activity	*Feel better in their bodies* *Gain benefits* *Connect with others* *Mothers experienced a kickstart into physical activity* *Mothers were challenged to maintain physical activity*

## Pre-program interviews

### Mothers were lost as to where to start physical activity

In trying to understand how mothers felt about physical activity after the birth of their child, analysis revealed that the birth experience impacted the way mothers are frustrated with physical activity, the way mothers view themselves, and the way mothers need help reclaiming a sense of agency and competency.

#### The way moms are frustrated with physical activity

Numerous participants described physical concerns from pregnancy or birth which were impacting their mobility and confidence in returning to physical activity. This included incision pain from caesarean sections and pelvic floor weakness from pregnancy and birth. Many participants described feeling weakness, inability to see progress in health and fitness, and a general sense of emotional distress transitioning to postpartum physical activity. For example, Brittany stated, “I’m feeling lost. I don’t really know where to start again.” Similar emotional frustration was expressed by Amanda who was “not able to do the things [I was] doing before.” Danielle also felt defeated due to not being “how fit I used to be,” which, combined with other mothers’ perspectives, was indicative of bodily capacity concerns surrounding changes to fitness and strength following delivery. Diana’s narrative directly captures this frustration with physical mobility limitations: “I find I have been having some pelvic floor weakness and so it’s stopped me from doing as much yoga just because I don’t feel as confident in it.”

While these physical changes were undoubtedly expected by mothers, common narratives of uncertainty and general impatience were revealing. After Jessica had her second child via caesarean section, she knew that her “body is not where it was,” and lamented the reality that “it takes time and there are certain stretches and things I have to implement in order to get my body there, but it’s such a lengthy process.” With Danielle, returning to physical activity “has been slower than expected” resulting in a general feeling of being unfit. Frustration and confusion resulted in feeling like Brittany was at “square one” with her fitness and “a little bit lost as to where to start again.” Lack of awareness of physical activity options, or simply unrealistic expectations evoked frustration for many, acutely observed by Elizabeth who shared, “I feel like I’m starting over again. So, when I do go for a run or something and everything is tight or like I can’t go as fast as I normally do, I get frustrated with myself.” Overall, evidence of frustration and emotional distress were seen in challenges recovering from pregnancy and birth, often related to participant expectations of their own fitness or physical activity capacity.

#### The way moms view themselves

Mothers’ inability to maintain expected or previous forms of physical activity further contributed to a growing sense of discomfort or a sense of feeling foreign within their own bodies. Hormonal changes, cultural pressure, as well as social media all influenced how mothers viewed themselves, with many expressing bodily dissatisfaction. Despite feeling well during the postpartum phase, Danielle “felt very foreign” in her body and maintained a negative attitude towards herself, while Elizabeth admitted to being “a little bit hard on myself where I want to be physically.” Mothers were not hesitant to share their bodily concerns, as Kimberly shared feeling “a little bit self-conscious” in group settings because of her “own self-image issues.” For many women, this feeling of disconnection to their postpartum body contributed to emotional distress, much like their frustrations towards physical activity in general. When Amanda considered her own bodily discomfort, it prompted a strong emotional reaction: “I feel upset sometimes … and sometimes I feel like, why did this happen? My body’s not the same.” When considering changes to her own body, Victoria felt “depressed about and grieving [my] old body,” which seems expressive of some dissociation with her current self. Perhaps, Sarah’s narrative best captures this inner tension between bodily acceptance and a desire to physically improve:

I just want to be present with my baby. I don’t want to be thinking about how my body looks…But every once in a while those thoughts creep in and it feels complicated. It feels complicated to know how to love my body but also want to change my body.

In addition to feeling a general sense of disconnection from their post-birth bodies, some women identified “silly expectations” to return to their pre-baby body; yet there was an emergent desire to “reclaim my body in a way” or experience a greater degree of agency over one’s body.

#### The way moms need help

As analysis revealed that mothers certainly struggled with managing expectations and maintaining healthy perspective, they additionally cited a number of barriers to physical activity, including child-minding, time constraints as well as general lack of energy or “the inertia of postpartum grubbiness” [Lisa]. Emotional fatigue aside, the sheer prospect of engaging in physical activity was often perceived as intimidating, as well-described by Stephanie: “It’s more difficult than I thought it would be … I never thought about all the things you would need to think of in order to make exercise possible.” Related to these barriers, mothers expressed a need for more help to get moving in the form of specific physical activity guidance and support. Specifically, mothers emphasized the need for expert guidance, program provision, personal prioritization, and social accountability to assist in maintaining physical activity engagement. For example, mothers expressed a desire to have guidance to “be told what to do and what not to do” [Lindsey] and to ensure “you’re doing exercise properly” [Diana] with a “hands on approach” [Danielle] to provide correction and feedback during movement. Group settings and the presence of others were viewed as facilitative of participation and accountability. For Ashley, broader program support would facilitate a more optimal postpartum experience:

I just think of how different our society would be if newly postpartum moms were held with more love and support. What that would do for changing families, raising happier and more stable children and what the spin-off of that effect would be on society as a whole. I think it’s profound. What we could experience a shift if new moms were supported better.

While motivation to engage in physical activity varied amongst the participants, most shared the need for education, guidance, and group support to begin or resume physical activity postpartum.

## Post-program interviews

Analysis revealed two primary themes within the post-intervention interviews, inclusive of data from the post-program as well as data from the 6-month follow-up interviews. First, engaging in physical activity helped the mothers. Within the first theme, three additional subthemes were named: the program helped the mothers a) feel better in their bodies; b) gain benefits and c) connect with others. Second, data showed that mothers learned to rethink their physical activity as supported by two subthemes: mothers a) experienced a kickstart into physical activity and b) were challenged to maintain physical activity.

### Engaging in physical activity helped mothers

The first primary theme identified through data analysis is that engaging in overall physical activity helped the mothers in various ways. Participants in the exercise intervention generally reported a positive and beneficial experience, even affirming the program as “the highlight of my day” [Amanda] and essential to helping them improve perceptions towards themselves and physical activity. For example, Nicole credited the program for affirming a renewed appreciation for health and well-being: “I sort of developed this kind of thinking that … my wellness is my baby’s wellness and so it felt really like a loving thing to do for me and my daughter when I’d go to the classes.” For Vanessa, who was historically less active, the program offered a fresh relief from previous experiences: “I never really thought that I’d look forward to physical activity … I have really looked forward to going every week, like anticipating to go. I’ve never had that before.” To fully appreciate how physical activity helped mothers, three subthemes were named. That is, the program helped mothers a) feel better in their bodies; b) gain benefits and c) connect with others.

#### Feel better in their bodies

Participation in physical activity helped mothers feel more capable and confident in their bodies as well as in their ability to be physically active. Most mothers attributed confidence improvements to fitness and strength development as well as learning how to move their bodies again. For example, Ashley shared that her experience in the program was transformative: “It was really intense, and it really challenged me … I’m capable of more than I give myself credit for … I’m so confident in my abilities more than anything.” Similarly, Sarah said participating in the program “improved my confidence because it showed me that I can be active again” and for others the program led to “feeling more confident in my body” [Victoria] as well as having more “confidence in my own movement” [Vanessa]. Even reflecting on the experience six months later, Diana shared how much her confidence grew, “I can do a lot more than I thought I could. I could handle and cope with a lot more mentally, physically … emotionally and stuff through like getting myself up, getting myself out the door with the baby, and all that kind of stuff to get to this workout.” Despite some initial uncertainties where Brittany “was a little bit afraid of trying certain things,” a renewed confidence in her ability emerged through being physically active. Improved confidence also stemmed from gaining a better understanding of what type of physical activity could be done after the birth of their child. New awareness included “knowing how much I can do,” [Brittany] having a more “open mind towards activity,” [Lauren} experiencing a sense that “I can do more” [Amanda]. Danielle, a first-time mother who had a history of being physically active, shared at length how she grew in confidence by realizing she could be active “with literally almost no equipment,” where the program activities “shifted my mindset … and learned how to do things differently for the body I have now.”

For Danielle and others, part of their increased confidence and capacity developed through their own improvements in physical fitness. For Stephanie, who questioned her fitness and abilities, the program provided physical health benefits: “I feel like I’m totally in shape, even though I’m only working out twice a week. It’s just made a huge difference physically. I feel stronger.” Mothers also experienced noticeable changes in their ability to complete the exercise classes, emphatically shared by Danielle who struggled in the first couple of weeks of the program:

I did struggle with being a little bit discouraged just because I did need to lower my expectations. I went in thinking, “It’s body weight stuff, I’ll be totally fine” … It was so hard at the beginning, and I was like, “Oh my god, I’m so unfit. This sucks.” But now I’m doing those classes and making it through the classes without having to take as many breaks, seeing that I have made a change.

The physical improvements were also experienced in activities of daily living where Elizabeth noted that getting up from a supine position had become easier. Several mothers also shared that their strength and fitness improvements were noticeable six months following the program. For example, Diana noticed “the couple months afterwards, after finishing [the program], I definitely felt a lot stronger … and able to go about my day a lot easier.” Despite becoming less active since completing the program, Lisa noted “certain pains that I had prior to doing the program” had not returned due to increases in physical fitness.

Another critical aspect of feeling better in their bodies, many mothers also shared how engaging in physical activity altered their perceptions and attitudes towards their bodies. Several mothers shared how they struggled with their bodies following the birth of their child. For example, Brittany described how the birth of her child brought her to “look at my body kind of differently” prompting emotional dissatisfaction given how “easy to get down on yourself.” However, participating in the program changed her outlook:

I think I was pretty discouraged, pretty down and just feeling like really self-conscious about myself … but [the program] helped my confidence in that regards where I just I feel good about myself, I feel like I can project a positive body image to [baby] just because I feel good.

Similarly, Danielle shared that, despite the difficulty in “getting used to the differences in the mirror,” engaging in physical activity helped her reframe how she views herself: “[Physical activity] is what’s gotten me better accepting my body and seeing little changes and knowing that it doesn’t have to stay this way forever. Even though it’s a slow process.” While participants indicated that a sense of self diminished during pregnancy, engaging in physical activity through the program helped restore that identity. In reflecting back on the program and being further along in her postpartum journey, Vanessa, a first-time mom, identified participating in the program as a major highlight in recovering her sense of self:

After having a baby, I felt like I would never recover and then completing the program … honestly like it just like gave me back what I felt I had lost … I’ve been thinking a lot about like this past year, and yeah, the classes were definitely a highlight of the year in terms of just, like, me feeling like myself again.

Overall, mothers reported that feeling “more toned and stronger” [Sarah], “getting back into our own body” [Nicole], as well as revaluing overall health was profound and helped them better accept the condition of their postpartum bodies.

#### Gain benefits

Engaging in physical activity also helped mothers gain mental health benefits. Participants described how engaging in physical activity helped take “bad moods away” [Ashley] reduced “feeling anxious or restless” [Willow], removed feelings of crankiness, led to feeling emotionally and mentally stronger, and, as Brittany summarized, “just lifted my overall mental health.” Some mothers expressed surprise at how much their mental health changed, Amanda sharing how she has felt more emotionally balanced since the start of the program: “I don’t think I’ve had any really bad emotional days since going into the program.” Similarly, Lisa commented on how her mental health prior to the program was of concern, but engaging in physical activity radically changed her treatment plan:

I haven’t had to go onto any medication for my mental health which … before this program started, my doctor suggested that I try that. But I figured that I would try this program a little bit first. It definitely has helped to the point where I haven’t had to do that.

Participating in physical activity through the program helped mothers “feel like you’re human again” [Elizabeth] provided opportunity to “focus on yourself” [Stephanie] and helped “build a bridge from where I am to where I want to be” [Nicole]. A sense of identity and self-determination, then, were core components of the benefits gained in being active. Crucially, those mental health benefits impacted other areas of their lives, including marriages, productivity around the home, better management of nutrition and sleep routines, and according to Kimberly, led her to be “less short, a little more patient throughout the day.” For Vanessa, the program provided a valuable outlet to prioritize self-care; in finding that motherhood blurs every day together where “you’re always getting up feeding the baby, changing the baby, making sure they’re happy and healthy,” physical activity provided opportunity to “look at myself again … and work on myself,” benefits that had been neglected due to child-rearing demands. Critically, 6-month follow-up data revealed that mothers who became less physically active also experienced deteriorated mental health. For example, Diana shared that she “let her physical activity go downhill” and as a result her “mental health isn’t really the greatest.” Similarly, Lisa shared that lack of regular physical activity resulted in not “feeling as great mentally.”

Another benefit emerging from programmed physical activity was the prioritization of self-care, or, as Vanessa shared, to make the time “to take care of myself.” An importance of self-care was expressed in a number of ways, such as “healing yourself”[Amanda], being “more involved in looking at things that I need to do in my own life for my health” [Brittany] and “feeling like I can make some progress on other things that aren’t my baby” [Nicole] Several self-care activities were mentioned by participants, including walking, counselling, healthy nutrition, as well as online support groups (e.g., self-compassion group). Many mothers highlighted the centrality of physical activity in maintaining health and a sense of stability and found that the program helped shape their perspective. For example, Nicole learned through the engaging in physical activity that:

Any exercise is better than no exercise and if it’s not an hour long, like a hardcore workout, you know, it’s still relevant. You know, like I did like 0 minutes of very low impact stuff today and it was like, I got a little sweat on, I felt amazing. I feel like a new woman just from that.

With the increase in importance placed on self care, mothers shared that the program shaped their perspectives of self and health, emphasizing the benefit of physical activity on their mental and emotional health.

#### Connect with others

Finally, a third subtheme that illustrates how physical activity helped the mothers is the connection that they experienced to other moms. The mothers in the program felt safe, included, and comfortable being with other women who were in a similar phase of life and this group connectedness increased their accountability to the program. For example, Amanda appreciated how the program helped mitigate her loneliness:

I’m not feeling bad about myself anymore. I’m not feeling lonely … It’s very motivating seeing other people doing things. And then everybody’s sharing their struggle as well. Not just the exercise part, [but] just being a new mother as well. Everybody’s sharing their things … So, just by talking to somebody in person, it makes you feel really good.

Many participants highlighted how the group experience led to “a sense of community,” where mothers were sharing stories, resources, and, in one instance, sharing breast milk to a mother who was experiencing insufficient supply. Danielle described the benefit in being active with other mothers due to her own challenges accepting her postpartum body:

Seeing different moms, with different postpartum bodies, like, some of them looking like they never had a baby. And then some of them who are more like me, where … you’ve got a little bit of the pouch, and a little bit of extra weight and knowing that you’re not the only one that struggles with that.

Mothers shared that they “met some really great people” [Stephanie] which led to additional meetups for walks and socialization outside of the program itself. However, continuity in maintaining those relationships varied, with Nicole noting in the 6-month interview that she was disappointed to lose touch: “I do feel like I ended up walking away from some budding friendships with other new moms that I had met in the group, and I lost touch with them.” The community of moms and women that were living similar postpartum experiences was a cherished memory of program participation.

Along with the sense of community, many women enjoyed that the program gave them a “sense of purpose” [Diana] and accountability to leave their house. The accountability to be active helped promote consistent routines for Nicole: “If I had been left to my own devices to return to exercise, I’m not sure I would have found my groove so quickly … Having a routine and the accountability of a group setting really, I think, accelerated the process.” Likewise, Brittany was motivated by guilt, sharing that “I’m way better being accountable to other people so I would always go to the workouts because I didn’t want to let anybody else down.” Once guilt dissipated upon program completion, however, the accountability to be physically active also dissipated. Danielle indicated that the group format helped motivate her participation in the exercise program:

Having someone there to be accountable to … I mean even the people that I didn’t know personally you kind of felt like, “Ah, they’re all going to be there … I gotta be there.” So, then they’re like, “Why didn’t you come on this day?” And so, I mean everybody understands when you’re like, “Oh, we had a rough night” … I think if it was an individual thing I would have a lot harder time getting myself there.

Overall, the accountability of joining the program led to increased purpose, motivation, and intentionality in getting out of the house which, for many participants, helped contribute to an overall sense of community and belonging.

### Mothers learned to rethink their physical activity

The second primary theme that the data revealed was that participating in the program led mothers to rethink their physical activity engagement. Rethinking their physical activity was inclusive of both facilitators and barriers to ongoing engagement. Therefore, the primary subthemes named within the overarching theme of rethinking physical activity includes that mothers a) experienced a kickstart into physical activity and b) were challenged to maintain physical activity.

#### Mothers experienced a kickstart into physical activity

For many mothers, the program served as a “kickstart to being more physically active” [Ashley]. Mothers shared that program participation helped them “make physical activity a priority,” [Jessica] and the program itself was associated with feeling “re-sparked … to do physical things,” [Danielle] and was “that little kickstart … a reboot to get back into it” [Lauren]. Regular participation helped Brittany get “back into being in love with being active again” and, for Nicole, the program “was such a critical steppingstone to get back to the activities I was doing before my pregnancy … a gentle re-entry to exercise in a safe and inclusive environment.” Simply participating in the program provided a new point of entry, and subsequent increased motivation, to promote physical activity engagement.

Beyond simply participating in the program itself, mothers shared they began to participate in more physical activity during the rest of the week. Specific activities included going for walks, working out at home, running, hiking and “just doing lots of active stuff on days off” [Brittany]. In developing a greater appreciation for overall well-being, including “making me want to do more things for my health”, the program influenced Sarah’s desire to continue with other physical activities, such as “look[ing] around for other types of fitness classes.” In addition, Nicole highlighted how the program provided the needed motivation boost to include her child within her physical activity:

Since going to the class, I’ve been super inspired about taking her on hikes with me, and you know, when she’s having some time on the floor, stretching next to her, doing a few push ups and kissing her on my way down. I’m finding a lot more integration between the two, whereas before these classes I kept thinking, like somehow it had to be separate, like I had to find a way to return to physical activity without her. But no, she’s my new appendage, she’s my new left arm.

Shifts in perspective also occurred for Diana who shared that, “I can tackle more with baby than I ever thought I could … now I’m kind of like, well whatever, he’s going to fuss, but I can get through it.” Likewise, Brittany shared her new ability to transfer her learning of engaging her child in her physical activity to her home environment: “I can do it with her crawling all over me and it’s not an issue or, you know, if I bring her into our yard and set up some toys, I can do some exercises outside too.” Therefore, some participants found the program provided a kickstart to engage in physical activity both in finding new physical activity pursuits as well as finding new ways to include their children within a physically active lifestyle.

Some specific aspects of the program were specifically identified as useful to shaping new perspectives towards physical activity. These included an inclusive environment with other mothers, an environment where children were welcomed and free to roam around, lack of pressure or expectation to be at a certain fitness level, and the general accountability that came with a structured and routine class. Nicole suggested such aspects are obvious, but nevertheless pivotal in supporting mothers: “A program like this for new moms to go to, it should exist. It should be a permanent fixture in the community, like free, accessible classes around other moms. It just seems like a no brainer.” Even after six months following the program, some mothers stated how they used program features to inform ongoing physical activity engagement. Some mothers started participating in online and app-based exercise programs, mimicked the intervention exercise routine at home, increased hiking and biking frequency, purchased gym memberships, and generally reported being more consistently physically active.

#### Mothers were challenged to maintain physical activity

While mothers generally indicated that the program informed how they view postpartum physical activity and kickstarted their physical activity engagement, some participants nevertheless highlighted various barriers to ongoing physical activity, especially in the 6-month follow-up interviews. Primary barriers included general fatigue or exhaustion, fussy, challenging, or highly mobile babies, and time constraints including return-to-work demands. Interestingly, while some mothers found it advantageous to include their child in their physical activity, others found it problematic. For example, Willow shared how her mental well-being was worse when her child accompanied her to the exercise program: “The ones where I had my baby with me, my anxiety was so much higher. Just from the logistics of getting out of the house with her. It was like, instead of showing up being like calmed and relaxed … I’m barely keeping it together.” At the 6-month follow-up interviews, many mothers further acknowledged increased challenge in being active now that their child was mobile and generally in need of significant attention. Victoria described how she always had to question whether her physical activity plan was feasible with her child or whether she would have to find childcare: “Can I do it with [baby]? Can somebody watch [baby]? What’s the best way to go about it?” Likewise, Kimberly shared that, “I can’t even imagine like trying to do any kind of activity … he just wants me to pick him up all the time.” The need to attend to a child led Lindsey to believe that, “I can’t do the proper workout. I can’t do what I actually need to do with those workouts, so it’s just not even worth it to do when she’s awake.” Therefore, despite learning the program how to include a child within their physical activity, increasing mobility within children continued to pose a significant barrier to continued physical activity.

Related to needing to attend to their child, participants specifically identified time as a substantial barrier to maintaining physical activity. Mothers expressed this barrier in a variety of ways, such as wishing there was “time to take care of myself” [Vanessa], simply “not having the time right now” [Ashley] and generally trying to balance the varied demands of day-to-day life. Brittany also found it difficult to find time for physical activity: “It’s hard for me to find the time, I guess. Because my preferred time to do a workout would be in the morning sometime … by the time the kids are both sleeping, I’m exhausted and, like just want to lie in bed and read.” One specific time constraint that mothers referenced in the 6-month interviews arose because several mothers had returned to work or were in the process of returning. This was noted as increasing exhaustion and further challenging mothers who had remained adherent to their physical activity goals. Jessica mentioned how much going back to work had benefitted her mental health but admitted that her ability to be active had been impacted: “I come home by the end of the day and I’m just like, exhausted cause I’ve been up with a kid all night, then work and then coming home and make dinner and doing stuff around the house.” Likewise, Lisa shared that “working has zapped some of my energy” which led to a reduction in physical activity. Between work life and home life, Sarah viewed exercise as an afterthought:

I think just finding the time and prioritizing it, especially when you’re tired and, like, with my work schedule, you know, work all night, and only sleep a couple hours and then watch her the rest of the day. It’s like the thought of exercising is just like, nope. That ain’t happening.

Overall, time management and life scheduling, then, posed a common barrier to physical activity engagement for several mothers. So, while mothers learned to rethink their physical activity during the program itself, some mothers continued to experience challenges in transferring those learnings into their life routines, especially once the structure delivery of the program completed.

## Discussion

Mothers who participated in this study were on average 6 months postpartum and were cleared to engage in physical activity by their physician. Prior to engaging in the exercise program mothers discussed challenges with physical activity frustrations, how they viewed themselves and their need for help in reclaiming a sense of agency over their lives and bodies. To resume physical activity postpartum, women noted that they needed support in becoming active. After engaging in the exercise program mothers felt better in their bodies and more capable engaging in physical activity, felt physically stronger, and were beginning to feel like themselves again. There was an increased sense of group support that led to accountability to the program. Women expressed numerous mental health benefits from their engagement in the program which contributed to a more positive body image. Generally, despite mixed capacity to navigate ongoing barriers to continued physical activity engagement, mothers shared that they learned to rethink their physical activity, including how to engage their child in their physical activity routine.

Our results parallel previous findings on physical activity engagement in postpartum women that have suggested complexity surrounding physical activity (Hamilton & White, 2010). Many of our mothers stated feeling foreign in their bodies and feeling like their body did not move like it used to. Frustrations with body comfort is in line with the common “bounce back” narrative as women are frequently in contact with imagery that reflects the societal expectation of the maternal body (Gow et al., 2012).Raspovic et al. (2020) interviewed women within the first 5 years of motherhood and found body image to be diverse, dynamic and individual. Mothers have been shown to have to both adjust to their new lives and bodies while attempting to negotiate the expectations of motherhood (Lloyd et al., 2016).

In addition, mothers expressed a lack of competence and self-efficacy surrounding postpartum physical activity engagement with concerns regarding their physical capacity. It is important to also note that over half of our mothers gave birth by caesarean section which may have a divergent recovery path compared to vaginal birth (Roy, 2014) and be further reflected in physical activity confidence. Liva et al. (2021) found that new mothers struggled to gauge appropriate physical activity engagement which led to over or underestimating personal capacity for physical activity. In post-program interviews immediately after our intervention, participants noted an increase in confidence in engaging in physical activity with a specific improvement in capability to be active with their baby. Given that many mothers found their baby to be the greatest hindrance to their ability to engage in physical activity before attending this exercise program, this is an important finding and suggests enhanced physical activity self-efficacy. There is a strong association between physical activity self-efficacy and physical activity engagement in postpartum women (Cramp & Bray, 2011). In addition, the guilt that is often associated with taking time to engage in physical activity pursuits within the expectations of motherhood (Bean & Wimbs, 2021), is reduced when the infant is part of the physical activity practice. The ability to engage in physical activity despite the barrier of having a child present is important for helping mothers fit physical activity into their busy lives, given that issues with childcare was a barrier among approximately one quarter of new mothers in the first year postpartum (Evenson et al., 2009).

Having followed up with these women six months post-program, it is interesting to note that despite increased confidence in engaging their baby in their physical activity, this had once again become a barrier as children became more mobile. This has been shown previously in new mothers, with physical activity barriers changing from 3 to 12-months postpartum due to the added challenge of a more mobile baby (Evenson et al., 2009). It is therefore important to continue to discuss ways to include children in physical activity programming over changes in growth and development.

Participants stated that the exercise program was a kickstart into physical activity though there was a range of longevity in physical activity practice, with some women finding it hard to continue to be active when the program ended, and others who continued or became more active. It appeared that our program was beneficial for improving physical activity self-efficacy in some mothers, demonstrated in mothers’ adoption of new physical activity practices and lifestyle integration. However, others were challenged in maintaining physical activity habits once the program finished largely due to exhaustion and a loss of accountability. Perhaps long-term adherence to physical activity would have been more achievable if mothers had learned self-regulatory strategies for overcoming barriers to physical activity engagement (Mailey & Hsu, 2019). Returning to work as a mother has been shown to lead to a loss of time for themselves as women navigated balancing motherhood and work commitments (Craig et al., 2012). In addition, barriers such as time and childcare were commonly noted among women that found it hard to continue to be active.

It has previously been shown that women in the postpartum transition require comprehensive care that enables new coping skills and adaptation (Ospina Romero et al., 2012). It appears that group-based physical activity programming may be a way of assisting new mothers with learning valuable skills to thrive in the postpartum period and may be in line with what has been shown to be of value to new mothers (Finlayson et al., 2020; Peralta et al., 2022). A qualitative study looking at group-based physical activity engagement of mothers with young children found that there was a requirement for: face-to-face sessions, convenient locations, appropriate timing, catering to babies and children, and a welcoming group environment to encourage continual physical activity (Peralta et al., 2022). This is further in line with our findings that the social and group connectedness of the program was highly valuable to participants. Social support is a vital component of the postpartum period and has been shown to be instrumental to mental well-being (Razurel et al., 2013). Women in the postpartum period commonly report inadequate social support (De Sousa Machado et al., 2020) and tend to seek support through other mothers (Strange et al., 2014). Therefore, group-based physical activity programs for postpartum women such as this one may provide the social support and the physical activity confidence needed to assist women in kickstarting physical activity after the birth of a child. One thing to note is that the exercise program utilized in this study, Les Mills Tone^TM^, is not designed specifically for postpartum women. Rather, this program was delivered showing low-impact options, and options for increasing and decreasing exercise intensity as needed. Thus, the findings of this study could be adapted to any group-based physical activity program.

There were many participants who noted improved mental health after engaging in the program. It is difficult to know whether this was due to the physical activity benefits, the benefits of being outdoors, or the social support, given that each of these are associated with mental well-being. Similar factors have been discussed in other physical activity interventions with new mothers where benefit to mental well-being may be borne out of physical activity and/or social support (Lee et al., 2016). Our study participants discussed the benefits of physical activity as being of value with getting stronger and fitter, further impacting their mental well-being due to a more positive outlook on their bodies. Our findings parallel the literature where mothers discuss the importance of focusing on the functionality of what the body can do rather than physical appearance (Peralta et al., 2021).

### Strengths and limitations

Consistent with qualitative description methodology, employed measures of rapport building, prolonged engagement at multiple time points, and ensuring the data thoroughly contained participant voices collectively support study robustness and trustworthiness (Finlay, 2006;Tong et al., 2007). Rigour and credibility were further supported by following an inductive thematic analysis process and ensuring thick description of participant experiences, essential in qualitative research (Tracy, 2010). In addition, employing a collaborative process amongst researchers throughout the analytic process mitigated researcher perception and subjectivity (Neergaard et al., 2009). Given the paucity of postpartum support, lack of opportunity for mothers to engage in physical activity, and low confidence surrounding physical activity engagement, findings from this study are relevant and timely (Tracy, 2010) and increase awareness of the experiences of new mothers engaging in physical activity.

Regarding study participants and process, several limitations can be noted. It is important to note that women who chose to participate in the exercise program were likely more physically active and confident than those who did not contact us to participate. New mothers who consider physical activity to be central to their well-being seek out physical activity options (Liva et al., 2021) and therefore create a voluntary bias. Future research therefore needs to expand recruitment to ensure that a broad population of postpartum women participate in specialized physical activity programming to determine its appropriateness. The lack of knowledge regarding physical activity levels of this population pre-pregnancy, during pregnancy and post pregnancy is a further limitation of this study as we cannot comment on the history and physical activity identity of our participants. Other studies have found that women may limit their engagement in mother and baby exercise classes if they were concerned about the environment not being inclusive (Liva et al., 2021; Peralta et al., 2022). Given the importance of group cohesion in group exercise dynamics, it may be important to develop postpartum physical activity groups based on similarity to promote inclusivity. In our study, almost all the mothers mentioned the likeability of the instructor being a factor in feeling like the environment was safe and inclusive. We recruited women who were 6 weeks through one-year postpartum to participate in our program. While there may be different dynamics at different stages of the postpartum period, it appears that barriers to physical activity are consistent throughout multiple time points in the postpartum period (Cramp & Bray, 2011). This study was conducted during the global COVID-19 pandemic and therefore mothers may have been experiencing enhanced mental duress and fewer opportunities for social support and physical activity programming.

### Conclusion and practical implications

Mothers who participate in a socially supportive group-based physical activity program may have better mental health outcomes and greater physical activity engagement. Health care providers should ascertain that postpartum mothers are provided access to the necessary education and programming around physical activity through referral or guidance. Recreation providers should consider offering programming specific to new mothers that allows for them to include their infant and include a supportive environment free of judgement. Additional findings from participant interviews were that programming for this population should be highly flexible with multiple times throughout the day due to changing demands with infant development. As well, greater emphasis should be placed on postpartum pelvic health in program design, a similar recommendation as found in Peralta et al. (2022) with mothers of young children. As demands on mothers rapidly change and many mothers return to work, future research should assess the benefits of providing ongoing physical activity support throughout postpartum and beyond.

Participants in this study expressed a lack of confidence in their bodies as well as in their capacity to engage in physical activity after the birth of a child. After engaging in a socially supportive group-based exercise program there was an increase in competence and motivation to engage in physical activity and engagement in this program was reflected in increased mental well-being. Six months after the program had ended, where many women discussed having returned to work, exhaustion and a loss of accountability made ongoing physical activity engagement challenging.
